# Draft genome sequence of *Listeria aquatica* strain SM_LAD isolated from cow dung in Bangladesh

**DOI:** 10.1128/mra.00938-25

**Published:** 2026-02-04

**Authors:** S. M. Mahfujur Rahman, Sangita Ahmed

**Affiliations:** 1 Department of Microbiology, University of Dhaka 95324, Dhaka, Bangladesh; University of Maryland Baltimore, Baltimore, Maryland, USA

**Keywords:** *Listeria aquatica*, Bangladesh, cowdung

## Abstract

The draft genome sequence of *Listeria aquatica* strain SM_LAD comprises 2,675,438 bp in 25 contigs with 2,674 coding sequences and a guanine and cytosine (GC) content of 39.54% and displays a virulence gene.

## ANNOUNCEMENT

*Listeria aquatica* is a non-pathogenic species first described in 2014 from freshwater environments in Florida, USA (1). It is considered a nonpathogenic species of *Listeria* genus, though studies report the presence of several putative genes of the pathogenic *L. monocytogenes* in this bacterium (2, 3). In Bangladesh, the prevalence of *Listeria* spp*.*, including *L. aquatica*, is apparent, but information about the genome sequence is negligible (4–6). This study reports on the draft genome sequence of an *L. aquatica* strain SM_LAD isolate isolated from cow dung in Bangladesh.

The cow dung sample collected from Gopalganj, Bangladesh (23° 19′ 0.12″ N 89° 52′ 0.12″ E) was aseptically placed into an ice box (4°C) and transferred within 12 h to the Biotechnology and Fermentation Laboratory of the University of Dhaka. The sample was then enriched in *Listeria* enrichment broth (Oxoid, England) for 48 h at 30°C, followed by streaking on *Listeria* selective agar (Oxoid, England) and incubation at 37°C for 48 h (7). A black colony from *Listeria* selective agar was selected for further identification by Gram staining and *prs* gene-specific PCR using primers F:5′-GCTGAAGAGATTGCGAAAGAAG-3′ and R:5′-CAAAGAAACCTTGGATTTGCGG-3′. Amplification was carried out with an initial denaturation at 95°C for 5 min, followed by 35 cycles of 94°C for 40 s, 57°C for 35 s, and 72°C for 40 s with a final extension at 72°C for 7 min (8). For whole-genome sequencing, genomic DNA was extracted by using the DNeasy Blood & Tissue Kit from an overnight culture of the isolate grown in tryptic soy broth and incubated at 37°C. Library preparation was performed at the icddr,b Genomics Center using the Illumina DNA Prep Kit, and sequencing was conducted on an Illumina NextSeq 2000 System generating 2 × 150 bp paired-end reads. No shearing or size selection of DNA was performed before the sequencing, and a total of 1,105,925 reads were generated for each sequence. Raw reads were quality filtered and trimmed using fastp (Galaxy Version 1.0.1) and assembled *de novo* with SPAdes (Galaxy Version 4.2.0), and genome annotation was performed using Prokka (Galaxy Version 1.14.6) and the National Center for Biotechnology Information (NCBI) PGAP (software revision: 6.10) (9, 10). Virulence and antibiotic resistance genes were detected by screening with CARD Resistance Gene Identifier (version 1.3.1) and ABRicate (Version 1.0.1) (11, 12). Default parameters were used for all software.

The final assembly parameters and genome features are mentioned in Table 1. The isolate was identified as *Listeria aquatica* using the One Codex database based on whole-genome sequence data (13).

**TABLE 1:** Genomic features of *Listeria aquatica* SM_LAD

Isolate ID	*Listeria aquatica* strain SM_LAD
Sample source	Cow dung
Year of isolation	2024
Genome length (bp)	2,675,438
No. of contigs	25
GC content (%)	39.54
N_50_ (bp)	285,851
Coverage (×)	78.35
Coding DNA sequence	2676
tRNA	44
rRNAs (5S, 16S, 23S)	3
Noncoding RNA	1
Antibiotic resistance gene	3 (*norC, vanT, vanY*)
Virulence gene	1 (*clpP*)
Accession no.	JBPOES000000000

## Data Availability

The whole-genome sequencing effort for *L. aquatica* strain SM_LAD has been submitted to GenBank with accession number JBPOES000000000 under the BioProject number PRJNA1273630 (BioSample accession number SAMN48952028 and SRA accession number SRS25765133). Additional details can be found here.
